# Aryl hydrocarbon receptor activation restores filaggrin expression via OVOL1 in atopic dermatitis

**DOI:** 10.1038/cddis.2017.322

**Published:** 2017-07-13

**Authors:** Gaku Tsuji, Akiko Hashimoto-Hachiya, Mari Kiyomatsu-Oda, Masaki Takemura, Fumitaka Ohno, Takamichi Ito, Saori Morino-Koga, Chikage Mitoma, Takeshi Nakahara, Hiroshi Uchi, Masutaka Furue

**Affiliations:** 1 Research and Clinical Center for Yusho and Dioxin, Kyushu University, Maidashi 3-1-1, Higashiku, Fukuoka 812-8582, Japan; 2 Department of Dermatology, Graduate School of Medical Sciences, Kyushu University, Maidashi 3-1-1, Higashiku, Fukuoka 812-8582, Japan; 3 Division of Skin Surface Sensing, Graduate School of Medical Sciences, Kyushu University, Maidashi 3-1-1, Higashiku, Fukuoka 812-8582, Japan

## Abstract

Filaggrin (*FLG*) mutation is a well-confirmed genetic aberration in atopic dermatitis (AD). Genome-wide association studies on AD have revealed other susceptibility genes, for example, Ovo-like 1 (OVOL1). Nonetheless, the relation between FLG and OVOL1 is unclear. Because aryl hydrocarbon receptor (AHR; a ligand-activated transcription factor), plays a role in *FLG* expression in keratinocytes, we hypothesized that AHR regulates FLG expression via OVOL1. To demonstrate this mechanism, we analyzed *FLG* expression in OVOL1-overexpressing or OVOL1-knockdown normal human epidermal keratinocytes (NHEKs). Furthermore, we tested whether AHR activation by 6-formylindolo(3,2-b)carbazole (FICZ), an endogenous AHR ligand, or Glyteer, clinically used soybean tar, upregulates FLG and OVOL1 expression in NHEKs. We found that (1) OVOL1 regulates FLG expression; (2) AHR activation upregulates OVOL1; and (3) AHR activation upregulates FLG via OVOL1. Moreover, nuclear translocation of OVOL1 was less pronounced in AD skin compared with normal skin. IL-4-treated NHEKs, an *in vitro* AD skin model, also showed inhibition of the OVOL1 nuclear translocation, which was restored by FICZ and Glyteer. Thus, targeting the AHR–OVOL1–FLG axis may provide new therapeutics for AD.

Mammalian epidermis comprises stratified squamous keratinocytes that protect the body from injuries by external (e.g., environmental) factors. Cornified envelope maturation is accomplished via sequential and coordinated cross-linking of ceramides and various skin barrier proteins, such as filaggrin (FLG), by transglutaminases 1 and 3.^1^ Recent studies revealed that disruption of the barrier function is crucial for the development of not only atopic dermatitis (AD) but also other allergic disorders, including asthma, allergic rhinitis, and food allergies.^2, 3^ Furthermore, a loss-of-function mutation of *FLG* is a well-confirmed genetic aberration associated with AD among different ethnic groups.^4, 5^ Therefore, clarifying the mechanism regulating *FLG* expression and establishing a strategy for increasing *FLG* expression may be beneficial for the treatment of AD.

Our recent studies have shown that activation of aryl hydrocarbon receptor (AHR), a ligand-activated transcription factor, is a key determinant of FLG expression in normal human epidermal keratinocytes (NHEKs).^6, 7^ Activated AHR relocates from the cytoplasm to nucleus, and this action induces transcription of the target genes, such as *CYP1A1*, in NHEKs. Nonetheless, the precise mechanism by which AHR regulates FLG expression remains unclear.

A series of genome-wide association studies conducted in Europe, China, and Japan have revealed other susceptibility genes, such as *OVOL1*, which is related to epidermal differentiation.^8, 9, 10, 11, 12, 13^ *OVOL1* is a ubiquitously conserved gene encoding a C2H2 zinc finger transcription factor in mammals. Functional studies in *Caenorhabditis elegans*, *Drosophila melanogaster*, and *Mus musculus* have suggested that this gene plays a pivotal role in the development of epithelial tissues arising from germ cells.^14, 15, 16^ Our studies and those conducted by other researchers have shown that OVOL1 is expressed in multiple somatic epithelial tissues, including human skin.^14, 17, 18^ Recent studies indicate that OVOL1 activation redirects cell proliferation to cell differentiation,^16, 19^ pointing to the possibility that OVOL1 controls the expression of skin barrier proteins, including FLG, during keratinocyte differentiation. Our recent study indicates that AHR activation by ketoconazole, a potent AHR ligand,^20^ upregulates *OVOL1* in NHEKs.^18^ Therefore, we hypothesized that AHR upregulates FLG via OVOL1 and that OVOL1 impairment is involved in FLG downregulation, which may possibly contribute to the development of AD.

To test this hypothesis, we analyzed FLG expression in OVOL1-overexpressing or OVOL1-knockdown NHEKs using the methods of electroporation and small interfering RNA (siRNA) transfection. Furthermore, to determine whether AHR activation upregulates or downregulates *OVOL1* and *FLG*, we used 6-formylindolo(3,2-b)carbazole (FICZ), an endogenous AHR ligand, and Glyteer, a soybean tar clinically used in Japan.

It has been already shown that AHR activation induced by coal tar increases FLG expression contributing to the therapeutic effect of coal tar on the development of AD;^21^ however, whether *OVOL1* is involved in the upregulation of FLG induced by AHR activation has not been examined. Herein, we demonstrate that *OVOL1* is an integral part for the AHR-mediated FLG expression in human keratinocytes.

## Results

**Figure 1:**
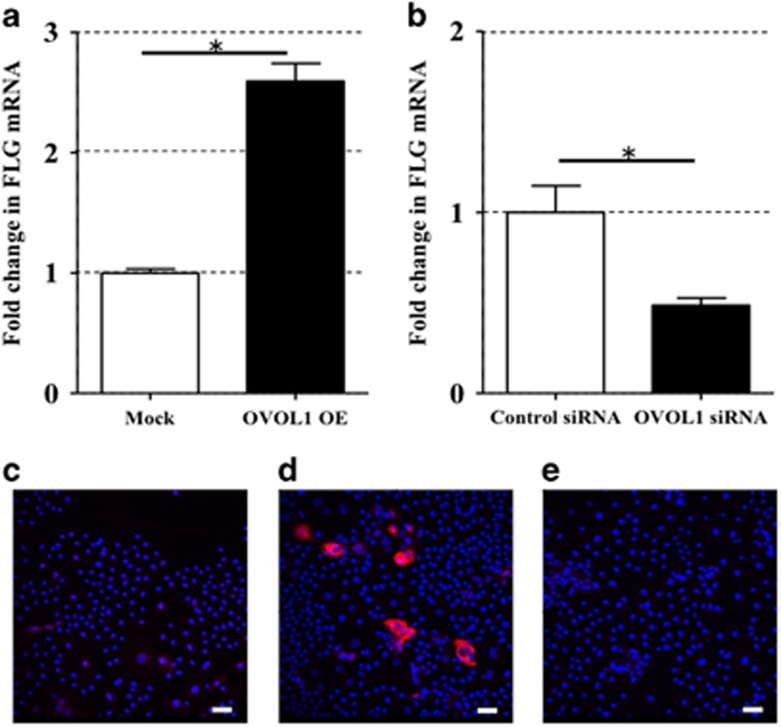
OVOL1 regulated FLG expression in NHEKs. (**a** and **b**) Data are expressed as mean±S.E.M.; *n*=3 for each group; *P<0.05. (**a**) OVOL1 was overexpressed in NHEKs (OVOL1 OE cells) by electroporation of the plasmid containing an open reading frame of human OVOL1. *FLG* expression in the OVOL1 OE cells was analyzed by qRT-PCR. (**b**) OVOL1 was knocked down by transfection of OVOL1 siRNA into NHEKs (OVOL1 siRNA cells). *FLG* expression in OVOL1 siRNA NHEKs was analyzed by qRT-PCR. Mock-transfected NHEKs (**c**) and OVOL1 OE NHEKs (**d**) were stained with an anti-FLG antibody (primary antibody) and an Alexa Fluor 546-conjugated anti-mouse IgG antibody (secondary). The nuclei were counterstained with DAPI (blue). Confocal laser scanning images revealed increased FLG expression (red) in OVOL1 OE NHEKs compared with mock-transfected NHEKs. (**e**) Isotype negative control. The scale bar is 25 *μ*m. The data are representative of experiments repeated three times with similar results

To examine the function of OVOL1 in *FLG* expression in NHEKs, we established either OVOL1-overexpressing (OVOL1 OE) NHEKs by electroporating the plasmid containing an open reading frame of human *OVOL1* into NHEKs or knocked OVOL1 down via transfection of OVOL1 siRNA. *FLG* expression was increased in OVOL1 OE NHEKs (Figure 1a). Conversely, *FLG* expression was significantly decreased in NHEKs transfected with OVOL1 siRNA (Figure 1b), indicating that OVOL1 is intimately involved in *FLG* expression in NHEKs. Compared with mock-transfected NHEKs (Figure 1c), the protein levels of FLG in OVOL1 OE NHEKs (Figure 1d) were increased according to immunofluorescence analysis with an anti-FLG antibody. The protein levels of OVOL1 either in OVOL1 OE NHEKs or OVOL1-knockdown NHEKs were evaluated by western blotting with an anti-OVOL1 antibody (Supplementary Figures S1A and B); this experiment confirmed that both types of transfection were successful. Next, to test whether OVOL1 regulates other genes of the epidermal differentiation complex including loricrin (*LOR*), involucrin (*IVL*), and transglutaminase 1 (*TGM1*) (representative terminal differentiation markers of keratinocytes), we analyzed *LOR*, *IVL*, and *TGM1* expression in OVOL1 OE NHEKs and OVOL1-knockdown NHEKs. In agreement with another report,^16^ *LOR*, but not *IVL* or *TGM1* expression was increased in OVOL1 OE NHEKs (Supplementary Figure S1C) and decreased in OVOL1-knockdown NHEKs (Supplementary Figure S1D). Therefore, OVOL1 specifically regulates *FLG* and *LOR* expression, contributing to the terminal differentiation of human keratinocytes.

Given that our previous studies revealed that AHR activation induces *FLG* expression in NHEKs,^6^ we determined whether AHR regulates the *OVOL1* expression in NHEKs. *OVOL1* expression was decreased in AHR-knockdown NHEKs transfected with AHR siRNA as compared with the cells transfected with control siRNA (Supplementary Figure S2A). The protein levels of AHR were evaluated by western blotting with an anti-AHR antibody (Supplementary Figure S2B); this assay confirmed that the knockdown of AHR by the transfection of siRNA against AHR was successful.

**Figure 2:**
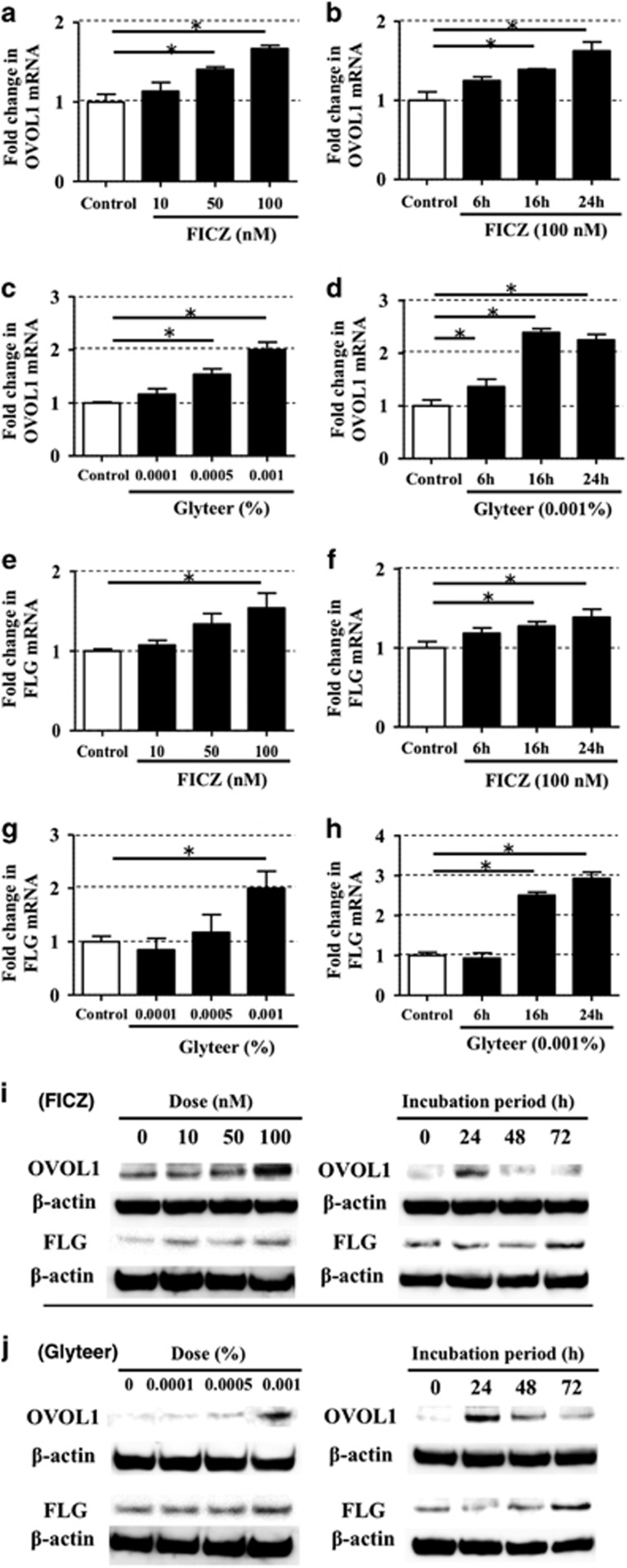
AHR activation induced by FICZ and Glyteer increased the expression of OVOL1 and FLG in a dose- and time-dependent manner in NHEKs. Data are expressed as mean±S.E.M.; *n*=3 for each group; *P<0.05. (**a**, **c**, **e**, and **g**) NHEKs were treated with FICZ or Glyteer at the indicated dose for 24 h. (**b**, **d**, **f**, and **h**) NHEKs were treated with FICZ (100 nM) or Glyteer (0.001%) for the indicated period. (**a**–**h**) Expression of *FLG* and *OVOL1* was analyzed by qRT-PCR. (**i** and **j**) NHEKs were treated with FICZ or Glyteer at the indicated dose for 24 h or for the indicated period. Total cell lysates were prepared and subjected to western blot analysis with an anti-FLG antibody and anti-OVOL1 antibody. The data are representative of experiments repeated three times with similar results

We next examined the effects of an AHR ligand on *OVOL1* and *FLG* expression. AHR activation by FICZ or Glyteer significantly upregulated *OVOL1* in a dose- and time-dependent manner (Figures 2a–d). The activation of AHR by FICZ or Glyteer also significantly increased *FLG* expression in a dose- and time-dependent manner (Figures 2e–h). Consistent with these results, AHR activation by FICZ or Glyteer increased OVOL1 and FLG expression in a dose- and time-dependent manner in a western blot analysis (Figures 2i and j).

**Figure 3:**
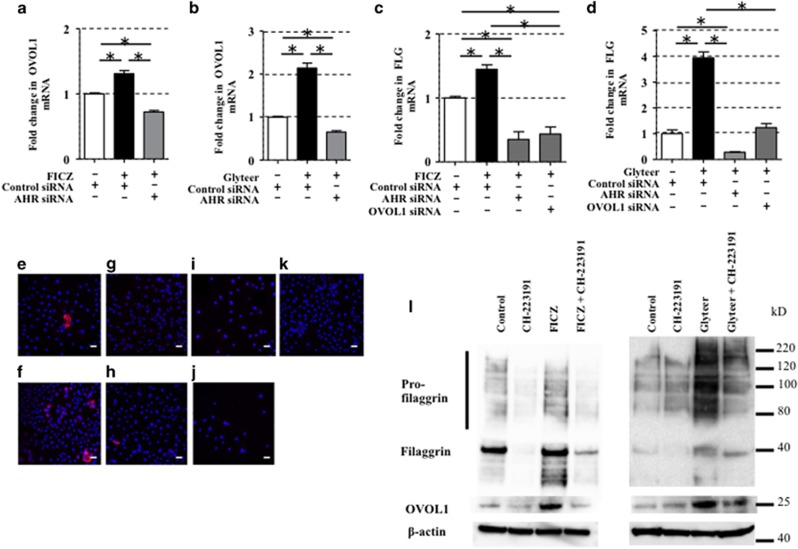
AHR regulated FLG expression via OVOL1 in NHEKs. (**a**–**d**) Data are expressed as mean±S.E.M.; *n*=3 for each group; *P<0.05. NHEKs were transfected with control siRNA or AHR siRNA and then treated with FICZ (100 nM) or Glyteer (0.001%) for 24 h. Expression of *OVOL1* and *FLG* in the NHEKs was analyzed by qRT-PCR. (**e**–**k**) NHEKs transfected with control siRNA, AHR siRNA, or OVOL1 siRNA were treated with DMSO or FICZ (100 nM) for 24 h and then stained with an anti-FLG antibody (primary antibody) and an Alexa Fluor 546-conjugated anti-mouse IgG antibody (secondary). The nuclei were counterstained with DAPI (blue). (**e**) Control siRNA-transfected NHEKs treated with DMSO, (**f**) control siRNA-transfected NHEKs treated with FICZ, (**g**) AHR siRNA-transfected NHEKs treated with DMSO, (**h**) AHR siRNA-transfected NHEKs treated with FICZ, (**i**) OVOL1 siRNA-transfected NHEKs treated with DMSO, and (**j**) OVOL1 siRNA-transfected NHEKs treated with FICZ. (**k**) Isotype negative control. Confocal laser scanning images revealed increased FLG expression (red) in control siRNA-transfected NHEKs treated with Glyteer (**f**) as compared with control siRNA-transfected NHEKs treated with DMSO (**e**); this upregulation was abrogated in AHR siRNA- or OVOL1 siRNA-transfected NHEKs treated with FICZ (**h** and **j**). The data are representative of experiments repeated three times with similar results. The scale bar is 25 *μ*m. (**l**) 3D-cultured NHEKs were treated with CH-223191 (10 *μ*M), FICZ (1 *μ*M), or CH-223191 (10 *μ*M) plus FICZ (1 *μ*M), or CH-223191 (10 *μ*M), Glyteer (0.01%), or CH-223191 (10 *μ*M) plus Glyteer (0.01%) for 48 h. Total cell lysates were prepared and subjected to western blot analysis with an anti-FLG antibody and an anti-OVOL1 antibody. The data are representative of experiments repeated three times with similar results

To test whether FICZ and Glyteer upregulate *OVOL1* and *FLG* via AHR, we analyzed FICZ- or Glyteer-treated NHEKs transfected with control siRNA or anti-AHR siRNA. The upregulation of *OVOL1* by FICZ (Figure 3a) and Glyteer (Figure 3b) was abrogated in AHR-knockdown NHEKs. Moreover, the upregulation of *FLG* by FICZ (Figure 3c) and Glyteer (Figure 3d) was abrogated in AHR-knockdown NHEKs and OVOL1-knockdown NHEKs. In the immunofluorescence analysis using the anti-FLG antibody, the upregulation of FLG induced by FICZ was abrogated in AHR-knockdown NHEKs and OVOL1-knockdown NHEKs (Figures 3e–j). Glyteer also showed the same pattern (Supplementary Figures S3A–C). These results indicate that FICZ and Glyteer upregulate *FLG* via the AHR−OVOL1 axis in NHEKs. Because the monolayer-cultured NHEKs system was difficult to use for evaluation of protein expression of FLG, including profilaggrin expression,^7^ we utilized three-dimensionally (3D) cultured NHEKs. In the western blot analysis, FICZ and Glyteer yielded increased OVOL1 and FLG protein expression, which was normalized by CH-223191, a specific antagonist of AHR (Figure 3l). This finding also confirmed that AHR activation increases OVOL1 and FLG protein levels.

**Figure 4:**
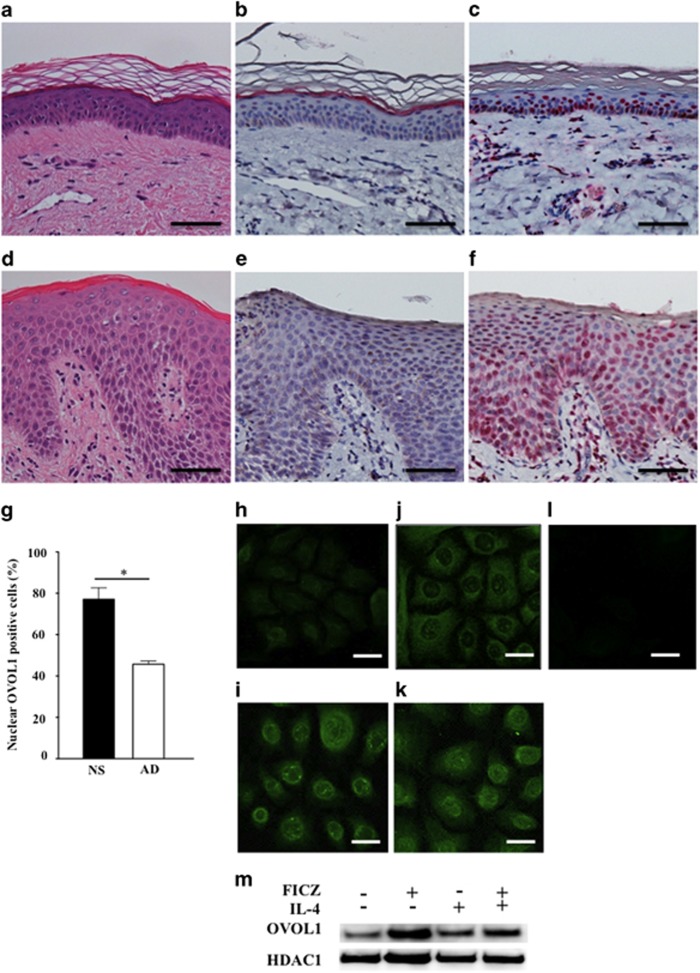
Nuclear translocation of OVOL1 was likely to be inhibited in AD skin, leading to the reduced FLG expression in AD skin. Normal skin (**a**) and AD skin (**d**) were stained with hematoxylin and eosin. The scale bar is 100 *μ*m. Expression of FLG and OVOL1 in the epidermis of the same skin lesion was analyzed by IHC staining for FLG (red) or OVOL1 (red). The expression of FLG was observed in normal skin (**b**) and was low in AD skin (**e**). The expression of OVOL1 was observed mainly in the nuclei of keratinocytes in normal skin (**c**); however, nuclear OVOL1 expression was lower in AD skin (**f**). For semiquantitative analysis of IHC staining, microscopic visual fields of the samples from each group were randomly chosen and examined. In a high-power field (× 400 magnification), the nuclear-OVOL1-stained cells of the epidermis were counted, as were all the cells with hematoxylin staining. Nuclear OVOL1 expression was lower in AD skin (AD) compared with normal skin (NS) (**g**). NHEKs treated with DMSO (**h**), FICZ (100 nM) (**i**), IL-4 (10 ng/ml) (**j**), or FICZ plus IL-4 (**k)** for 24 h were stained with an anti-OVOL1 antibody (primary antibody) and an Alexa Fluor 488-conjugated anti-rabbit IgG antibody (secondary). The nuclei were counterstained with DAPI (blue). Confocal laser scanning images revealed that OVOL1 expression was noticeable mainly in the cytoplasm in a steady state (**h**) and that the AHR activation by FICZ induced nuclear translocation of OVOL1 (**i**). In contrast, IL-4 did not induce nuclear translocation of OVOL1, and the latter was retained in the cytoplasm (**j**). IL-4-mediated blockade of the nuclear translocation of OVOL1 was overridden by treatment with FICZ (**k**). (**l**) Isotype negative control. The scale bar is 25 *μ*m. The data are representative of experiments repeated three times with similar results. (**m**) NHEKs were treated with FICZ (100 nM) in the absence or presence of IL-4 (10 ng/ml) for 18 h. Cellular nuclear protein was extracted using a biochemical subcellular fractionation technique. The OVOL1 levels in the nuclear protein fraction of NHEKs were evaluated by western blotting. The activation of AHR by FICZ increased the nuclear OVOL1 expression; in contrast, IL-4 did not change nuclear expression of OVOL1. The IL-4-mediated blockade of the OVOL1 nuclear translocation was partially reversed by treatment with FICZ. The data are representative of experiments repeated three times with similar results

To identify the role of OVOL1 and FLG in the AD pathogenesis, we evaluated OVOL1 and FLG expression in clinical samples of either normal skin or AD skin by immunohistochemical (IHC) staining. Compared with normal skin (Figures 4a and b), FLG expression was lower in AD skin (Figures 4d and e); this finding is consistent with another study.^22^

The expression of OVOL1 was observed mainly in the nucleus of keratinocytes in normal skin (Figure 4c); however, OVOL1 was not expressed in the FLG-positive cells in normal skin. As shown in Figures 2i and j, the FICZ- and Glyteer- induced upregulation of OVOL1 expression peaked at 24 h and gradually diminished until 48 h. In contrast, the FICZ- and Glyteer-induced FLG expression was observed to increase at 72 h. Therefore, the gap between the expression levels of OVOL1 and FLG in keratinocytes in the IHC staining experiment presumably resulted from the lag of the expression of OVOL1 and FLG in human keratinocytes.

The nuclear OVOL1 expression was lower in AD skin (Figures 4f and g) compared with normal skin, suggesting that nuclear translocation of OVOL1 is likely to be inhibited in AD skin. Because OVOL1 performs a transcription-regulatory function in the nucleus,^18^ these results suggest that the transcription-regulatory activity of OVOL1 may be impaired in AD skin, subsequently leading to the reduced FLG expression in AD skin.

To identify the mechanism by which the OVOL1 nuclear translocation is inhibited in AD skin, we analyzed OVOL1 expression in NHEKs treated with IL-4, a powerful suppressor of FLG expression; treatment with IL-4 can serve as an *in vitro* AD model.^7, 21^ Our previous study has shown that steady-state OVOL1 expression is primarily present in the cytoplasm of NHEKs.^18^ Ketoconazole, a potent AHR ligand, induces nuclear translocation of OVOL1 in NHEKs.^18^ Therefore, we used immunofluorescence analysis to test whether IL-4 affects the cytoplasm-to-nucleus translocation of OVOL1 in NHEKs. OVOL1 was stained with an anti-OVOL1 antibody (primary antibody) and an Alexa Fluor 488-conjugated anti-rabbit IgG antibody (secondary), which emits bright green fluorescence. 4′,6-Diamidino-2-phenylindole (DAPI), which emits blue fluorescence, was used for nuclear counterstaining. We determined whether the two-color fluorescent signals (green and blue) coincided in the nucleus to detect the OVOL1 nuclear translocation. In baseline NHEKs, OVOL1 expression was noted mainly in the cytoplasm (Figure 4h). Just as ketoconazole, the activation of AHR by FICZ clearly induced the nuclear translocation of OVOL1 (Figure 4i). In contrast, IL-4 did not induce OVOL1 nuclear translocation, and OVOL1 was retained in the cytoplasm (Figure 4j). IL-4-mediated blockade of the OVOL1 nuclear translocation was overcome by treatment with FICZ (Figure 4k). Similar results were obtained with Glyteer (Supplementary Figures S4A and B). These results were confirmed by western blot analysis. NHEKs were treated with FICZ (100 nM) or Glyteer (0.001%) in the absence or presence of IL-4 (10 ng/ml) for 18 h. Cell nuclear protein was extracted using a biochemical subcellular fractionation technique. The OVOL1 levels in the nuclear protein fraction of NHEKs were evaluated by western blotting. Histone deacetylase 1 (HDAC1) served as an internal loading control. The activation of AHR by FICZ increased the nuclear OVOL1 expression; in contrast, IL-4 did not change nuclear expression of OVOL1. The IL-4-mediated blockade of the OVOL1 nuclear translocation was partially reversed by treatment with FICZ (Figure 4m). A similar result was obtained with Glyteer (Supplementary Figure S4C).

**Figure 5:**
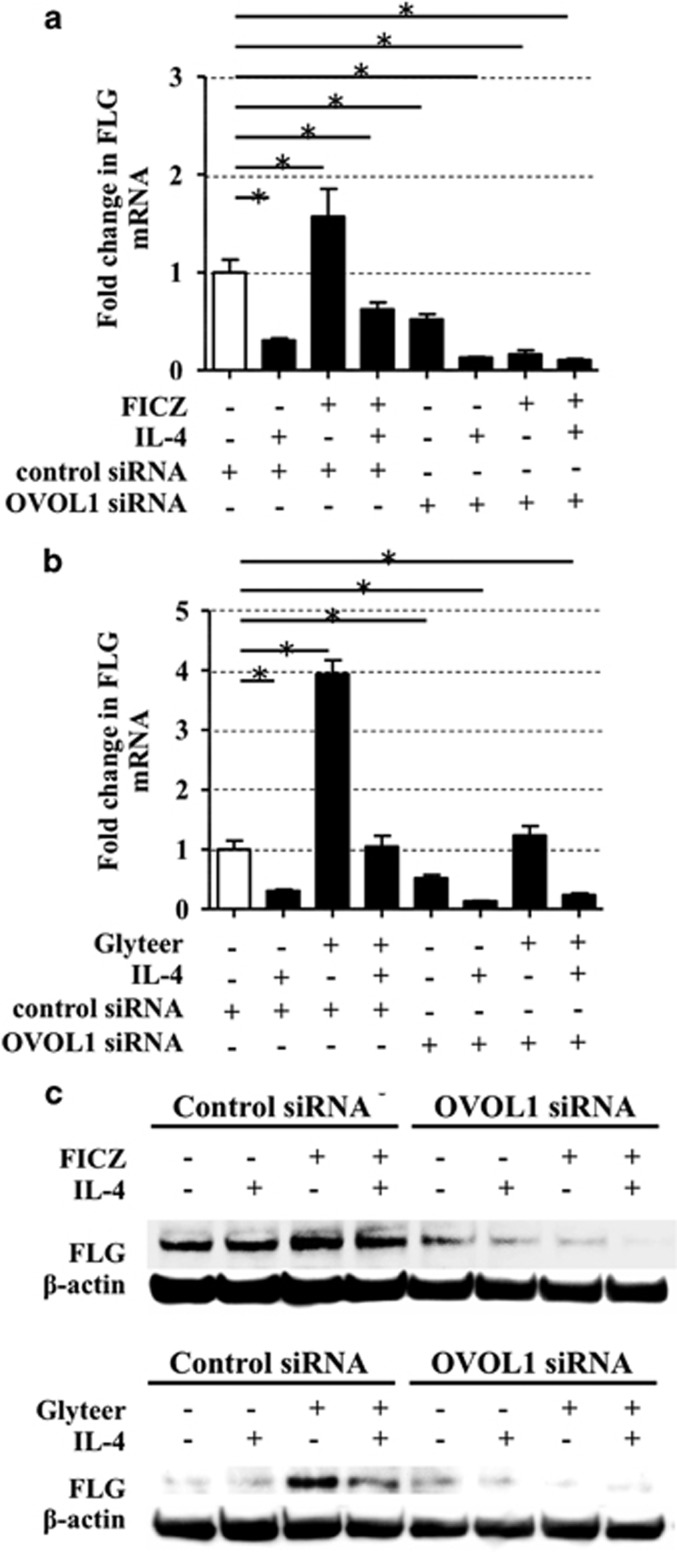
FICZ and Glyteer reversed the IL-4-induced decrease in FLG expression, which was dependent on OVOL1. Control siRNA- or OVOL1 siRNA-transfected NHEKs were treated with FICZ (100 nM) (**a**) or Glyteer (0.001%) (**b**) with or without IL-4 (10 ng/ml) for 24 h and then mRNA or total protein of the NHEKs were extracted. Expression of *FLG* in the NHEKs was analyzed by qRT-PCR. Data are expressed as mean±S.E.M.; *n*=3 for each group; *P<0.05. (**c**) Expression of FLG was analyzed by western blotting using the anti-FLG antibody. The data are representative of experiments repeated three times with similar results. The ability of FICZ or Glyteer to reverse the IL-4-induced downregulation of FLG was abrogated in OVOL1-knockdown NHEKs

In line with these results, FICZ and Glyteer reversed the IL-4-induced FLG downregulation in mRNA levels (Figures 5a and b) and protein levels (Figure 5c). To further elucidate whether OVOL1 is involved in the IL-4-induced *FLG* downregulation, we analyzed OVOL1-knockdown NHEKs treated with FICZ or Glyteer plus IL-4. The ability of FICZ or Glyteer to reverse the IL-4-induced FLG downregulation was abrogated in OVOL1-knockdown NHEKs (Figures 5a–c). We hypothesized that IL-4 inhibits *OVOL1* expression in NHEKs; however, this was not the case. Rather, IL-4 increased the baseline, FICZ-induced, or Glyteer-induced *OVOL1* expression (Supplementary Figure S5).

## Discussion

*Ovol1* has been shown to be a transcriptional factor important for the expression of murine epidermal differentiation complex genes, including *Flg*, *Ivl*, and *Lor*.^16, 23^ Furthermore, genetic depletion of *Ovol1* results in downregulation of *Flg* expression in murine keratinocytes.^23^ Therefore, *Ovol1* possibly regulates the expression of *Flg* in murine keratinocytes transcriptionally; however, the precise mechanism in human keratinocytes has not been fully examined.

In agreement with these findings, our previous study showed that OVOL1 and OVOL2 are preferentially expressed in human keratinocytes, indicating that the OVOL1–OVOL2 axis coordinately controls human keratinocyte differentiation.^18^ In the present study, we demonstrated that *OVOL1* is an integral part of the AHR-mediated mechanism of FLG expression in human keratinocytes.

The finding that *OVOL1* positively regulates *FLG* expression provides an important insight into the known phenomenon of reduced *FLG* expression in AD patients. Recent genome-wide association studies on AD have identified three single-nucleotide polymorphisms (SNPs), rs479844 (*OVOL1*), rs3126085 (*FLG*), and rs11204971 (*FLG*), that are associated with AD in Japanese and Chinese patients.^8, 9, 10, 11, 12, 13^ The A allele of rs479844 (*OVOL1*) reportedly reduces AD risk.^24^ Furthermore, *FLG* mutation or dysfunction is a crucial factor in AD development.^4, 5^ Therefore, we can hypothesize that OVOL1 impairment results in the downregulation of FLG, thus potentiating the development of AD.

We and other researchers have demonstrated that tar derived from coal or soybeans clinically used in Japan upregulates *FLG* via AHR activation, contributing to improvement of the barrier function in AD.^7, 21^ It has been shown in AHR knockdown NHEKs^21^ that an AHR-binding site (xenobiotic response element) in the *FLG* promoter region has an important role in the upregulation of FLG induced by AHR activation. Nevertheless, we next revealed that the upregulation of FLG induced by AHR activation was abrogated in either AHR knockdown or OVOL1-knockdown NHEKs.

**Figure 6:**
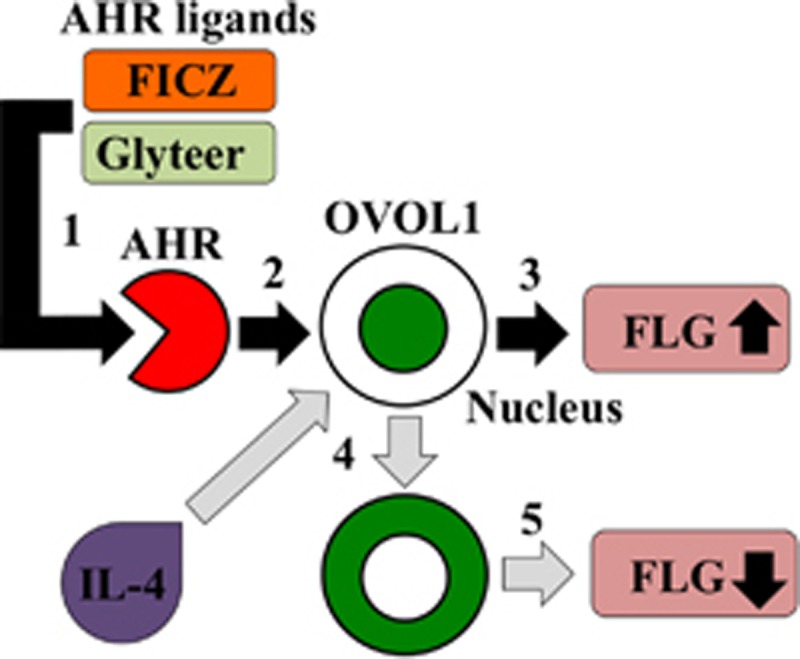
AHR activation induced by FICZ and Glyteer restores FLG expression via OVOL1 in the skin affected by atopic dermatitis (AD). 1. FICZ and Glyteer bind to AHR thereby leading to AHR activation. 2. Activated AHR induces upregulation and nuclear translocation of OVOL1. 3. The OVOL1 nuclear translocation of OVOL1 increases FLG expression. 4. IL-4 inhibits the nuclear translocation of OVOL1. 5. Inhibition of the nuclear translocation of OVOL1 downregulates FLG

We found that the *OVOL1* promoter between genomic positions 65786247 and 65787182 (NCBI Reference Sequence: NC_000011.10) contains three ‘GCGTG’ (xenobiotic response element) sites. This notion is in agreement with the fact that ketoconazole, an AHR activator, increases *OVOL1* expression in NHEKs, underscoring the possibility that AHR transcriptionally mediates *OVOL1* expression in NHEKs. The present study clearly showed that OVOL1 levels correlate with FLG mRNA and protein levels and that AHR-mediated FLG upregulation is abrogated in OVOL1-knockdown NHEKs. To further characterize the involvement of OVOL1 in the pathogenesis of AD, we analyzed OVOL1 expression in clinical samples of AD skin and in IL-4-treated NHEKs. To the best of our knowledge, here we for the first time showed that nuclear translocation of OVOL1 is inhibited in AD skin. On the basis of these results, we assumed that the blockade of OVOL1 nuclear translocation may be responsible for the inhibitory action of IL-4 on *FLG* expression. The activation of AHR by FICZ and Glyteer reversed the IL-4-induced blockade and facilitated the cytoplasm-to-nucleus translocation of OVOL1, thereby increasing *FLG* transcription (Figure 6). Given that FICZ, a photoproduct of tryptophan, is generated by ultraviolet irradiation in the skin,^25^ it is possible that phototherapy improves the skin condition in AD via the AHR–OVOL1–FLG pathway. Nevertheless, the precise mechanism by which IL-4 inhibits the nuclear translocation of OVOL1 remains unclear, and further studies are needed.

Thus, we demonstrated that OVOL1 positively controls FLG expression and that AHR regulates FLG expression via OVOL1 in NHEKs. These results suggest that a potent AHR–OVOL1 activator may have a therapeutic potential in AD via upregulation of *FLG*.

## Materials and Methods

### Reagents and antibodies

FICZ was purchased from Enzo Life Sciences (Exeter, UK). Glyteer was provided as an original stock solution by Fujinaga Pharm Co. Ltd. (Tokyo, Japan). DMSO and CH-223191 were purchased from Sigma-Aldrich (St. Louis, MO). IL-4 was purchased from R&D Systems (Minneapolis, MN, USA). For western blotting analysis, an anti-AHR rabbit polyclonal IgG antibody (Santa Cruz Biotechnology, Dallas, TX, USA), anti-OVOL1 mouse monoclonal antibody (Abcam, Cambridge, UK), and anti-FLG mouse antibody (Santa Cruz Biotechnology) were used. For immunofluorescent staining and IHC analysis, an anti-OVOL1 rabbit polyclonal antibody (LifeSpan BioSciences, Inc., Seattle, WA, USA) and anti-FLG mouse monoclonal antibody (Abcam) were applied. Normal rabbit IgG was purchased from Santa Cruz Biotechnology.

### Cell culture

NHEKs obtained from Clonetics-BioWhittaker (San Diego, CA, USA) were grown in culture dishes at 37 °C and 5% CO_2_. NHEKs were cultured in a serum-free keratinocyte growth medium (Lonza, Walkersville, MD, USA) supplemented with bovine pituitary extract, recombinant epidermal growth factor, insulin, hydrocortisone, transferrin, and epinephrine. The culture medium was replaced every 2 days. Near-confluent (70–90%) cells were disaggregated with 0.25 mg/ml trypsin/0.01% ethylenediamine tetraacetic acid and subcultured. Second- to fourth-passage NHEKs were used in all the experiments.

NHEKs (10^5^) were seeded in 24-well culture plates, allowed to attach for 24 h, and then incubated with or without FICZ, Glyteer, DMSO, and/or IL-4. Various concentrations of FICZ (10–100 nM), Glyteer (0.0001–0.001%), DMSO, and IL-4 (10 ng/ml) were prepared in the cell culture medium.

### Transfection of siRNAs against AHR and OVOL1

siRNAs against AHR (AHR siRNA, s1200) or OVOL1 (OVOL1 siRNA, s9939) as well as siRNA consisting of a scrambled sequence that would not lead to specific degradation of any cellular mRNA (control siRNA) were purchased from Ambion (Austin, TX, USA). NHEKs cultured in 24-well plates were incubated for 48 h in 0.5 ml of the culture medium with a mixture containing 5 nM siRNA and 3 *μ*l of the HiPerFect Transfection reagent (Qiagen, Courtaboeuf, France).

### Plasmid DNA and transfection of plasmids

Plasmids pCMV6-Entry (Mock) and OVOL1 (Myc-DDK-tagged)-Human ovo-like 1(Drosophila) (OVOL1), which contains a cytomegalovirus promoter and OVOL1 (NM_004561) human cDNA open reading frame clone, were obtained from Origene (Rockville, MD, USA). The plasmids were transfected into NHEKs using 4D-Nucleofector (Lonza, Basel, Switzerland).

### Quantitative reverse transcription-PCR analysis

Total RNA was extracted using the RNeasy Mini kit (Qiagen). Reverse transcription was performed using PrimeScript RT reagent kit (Takara Bio, Otsu, Japan). Quantitative reverse transcription (qRT)-PCR was conducted on a CFX Connect Real-time System (Bio-Rad, Hercules, CA, USA) using SYBR Premix Ex Taq (Takara Bio). Amplification was initiated at 95 °C for 30 s as the first step, followed by 40 cycles of qRT-PCR at 95 °C for 5 s and at 60 °C for 20 s. mRNA expression was measured in triplicate and mRNA levels normalized to *β*-actin were expressed as fold induction relative to the control group. The sequences of primers from Takara Bio are presented in the Supplementary Figure S6.

### Immunofluorescent staining and confocal laser scanning microscopy

NHEKs (2 × 10^4^) cultured on slides (Lab-Tek, Rochester, NY, USA) with or without IL-4, FICZ, and/or Glyteer were washed in phosphate-buffered saline (PBS), fixed with acetone for 10 min, and blocked using 10% bovine serum albumin in PBS for 30 min. The samples were incubated with an anti-FLG antibody or anti-OVOL1 antibody in WesternBreeze Blocker/Diluent (Invitrogen, Carlsbad, CA, USA) overnight at 4 °C. The slides were washed with PBS before incubation for 1 h at room temperature with a secondary antibody: an anti-rabbit IgG or anti-mouse IgG antibody (conjugated with Alexa Fluor 488 or Alexa Fluor 546; Molecular Probes, Eugene, OR, USA). After nuclear staining with DAPI, which emits blue fluorescence, slides were mounted with the UltraCruz Mounting Medium (Santa Cruz Biotechnology). The nuclear translocation of OVOL1 in NHEKs was evaluated by detection of the coincidence of the two-color fluorescence (green and blue) in the nucleus. All the samples were analyzed using a D-Eclipse confocal laser scanning microscope (Nikon, Tokyo, Japan).

### IHC analysis of normal skin and AD skin

This experiment was conducted in accordance with the principles embodied in the Declaration of Helsinki, and was approved by the institutional Ethics Committee.

Ten AD skin samples from 10 AD patients and 10 normal skin samples were used. All the samples were obtained from the archives of the Department of Dermatology of Kyushu University Hospital, Japan. Clinical and demographic data retrieved from the patient files were consistent with a diagnosis of AD.

All samples were fixed with 10% buffered formalin. The archival paraffin-embedded tissue blocks were cut into 4-*μ*m-thick tissue slices. The slices were deparaffinized with xylene for 10 min and rehydrated by means of a graded ethanol series. Antigen retrieval was performed using the Heat Processor Solution pH 6 (Nichirei Biosciences Inc., Tokyo, Japan) at 100 °C for 40 min. The slices were then incubated with an anti-FLG antibody at room temperature for 1 h or an anti-OVOL1 antibody at 4 °C overnight, followed by incubation with a secondary antibody, N-Histofine Simple Stain AP (Nichirei Biosciences Inc.). Immunodetection was conducted with Fast Red (Nichirei Biosciences Inc.), followed by light counterstaining with hematoxylin. The slices stained without a primary antibody served as a negative control.

For semiquantitative analysis of IHC staining, microscopic visual fields of the samples from each group were randomly chosen and examined. In a high-power field (× 400 magnification), the nuclear-OVOL1-stained cells of the epidermis were counted, as was the total number of cells with hematoxylin staining.

### 3D-cultured NHEKs as human skin equivalents

The human epidermal 3D model (EpiDem EPI-200: MatTek, Ashland, MA, USA) was incubated with or without Glyteer (0.01%), FICZ (1 *μ*M), and/or CH-223191 (10 *μ*M) for 48 h at 37 °C.

### Western blotting

Cells were incubated for 5 min in lysis buffer (Complete lysis M; Roche Diagnostics, Basel, Switzerland). The protein concentration in the lysate was measured using a BCA Protein Assay Kit (Thermo Fisher Scientific, Rockford, IL, USA). Equal amounts of protein (20 *μ*g) were dissolved in NuPAGE LDS sample buffer (Invitrogen) and a 10% sample reducing agent (Invitrogen). The lysates were boiled at 70 °C for 10 min and then loaded into and subjected to electrophoresis in NuPAGE 4–12% Bis-Tris gels (Invitrogen) at 200 V for 60 min. The proteins were then transferred onto polyvinylidene difluoride membranes (Invitrogen), and the membranes were blocked with WesternBreeze Blocker/Diluent (Invitrogen). The membranes were then probed with the anti-FLG antibody, anti-OVOL1 antibody, and a mouse monoclonal antibody against human *β*-actin (anti-*β*-actin) (Cell Signaling Technology, Danvers, MA, USA) overnight at 4 °C. Horseradish peroxidase-conjugated anti-mouse IgG antibodies (Cell Signaling Technology) served as a secondary antibody. The visualization of protein bands was accomplished with the SuperSignal West Pico Chemiluminescent Substrate (Thermo Scientific) by ChemiDoc touch imaging system (Bio-Rad).

### Cellular nuclear protein preparation for western blot analysis

NHEKs were treated with FICZ (100 nM) or Glyteer (0.001%) in the absence or presence of IL-4 (10 ng/ml) for 18 h. Cell nuclear protein was collected using NE-PER Nuclear and Cytoplasmic Extraction Reagents (Thermo Fisher Scientific, Rockford, IL, USA). The nuclear OVOL1 expression in NHEKs was analyzed by western blotting. Histone deacetylase 1 (HDAC1) served as an internal loading control. An anti-human HDCA1 antibody was purchased from Cell Signaling Technology.

### Statistical analysis

Unpaired Student’s *t*-test or one-way analysis of variance was used to assess the results. A *P-*value of <0.05 was assumed to indicate a statistically significant difference. All data are presented as mean±standard error of the mean (S.E.M.) from three independent experiments.
