# Supplementary figures and images for: Aryl hydrocarbon receptor activation restores filaggrin expression via OVOL1 in atopic dermatitis
Source: Cell Death Dis. 2017 Jul 13;8(7):e2931–. doi: 10.1038/cddis.2017.322 (PMC5550867; doi:10.1038/cddis.2017.322)

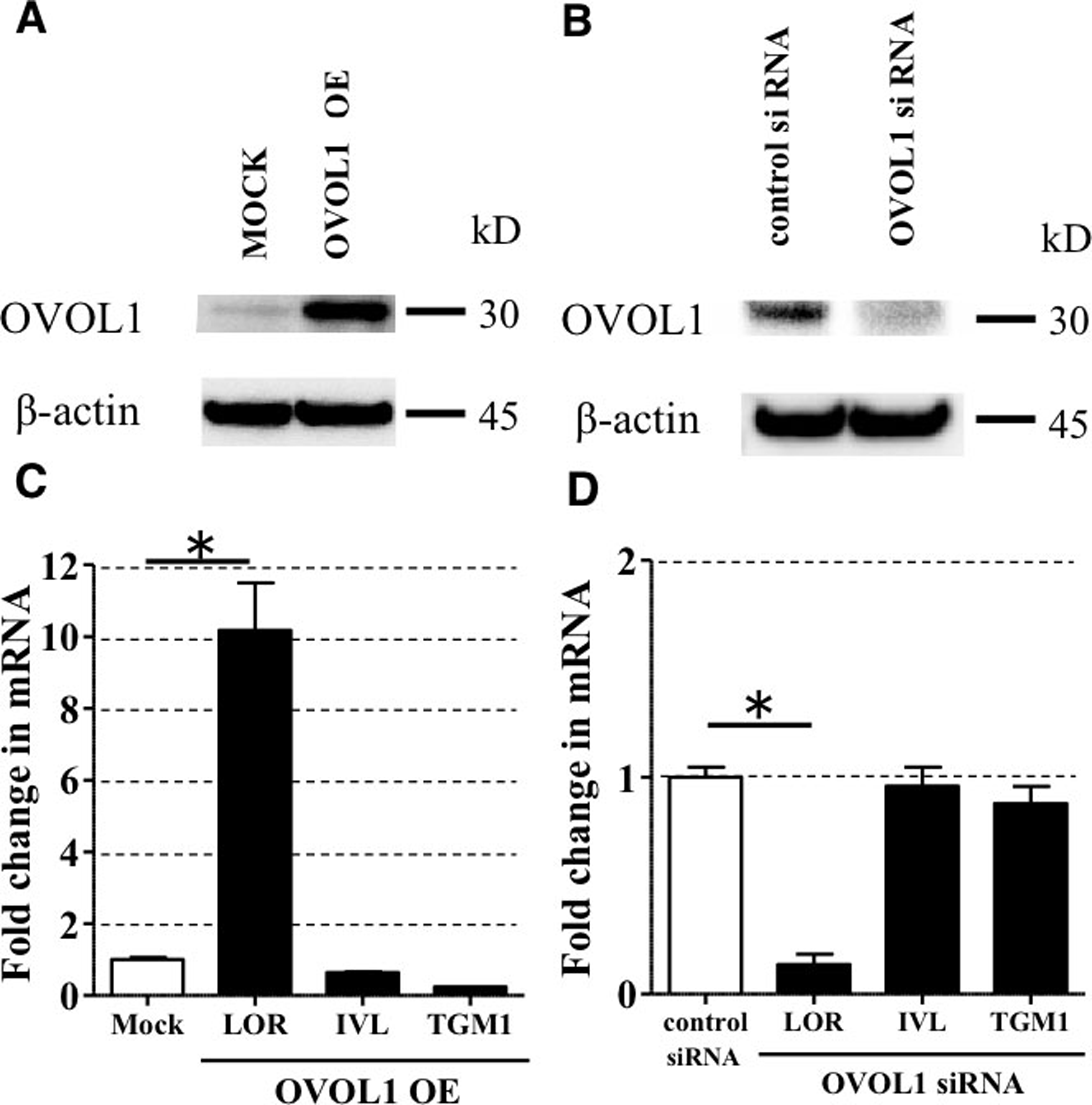

Supplement: Supplementary Figure S1 [file cddis2017322x1.tif]

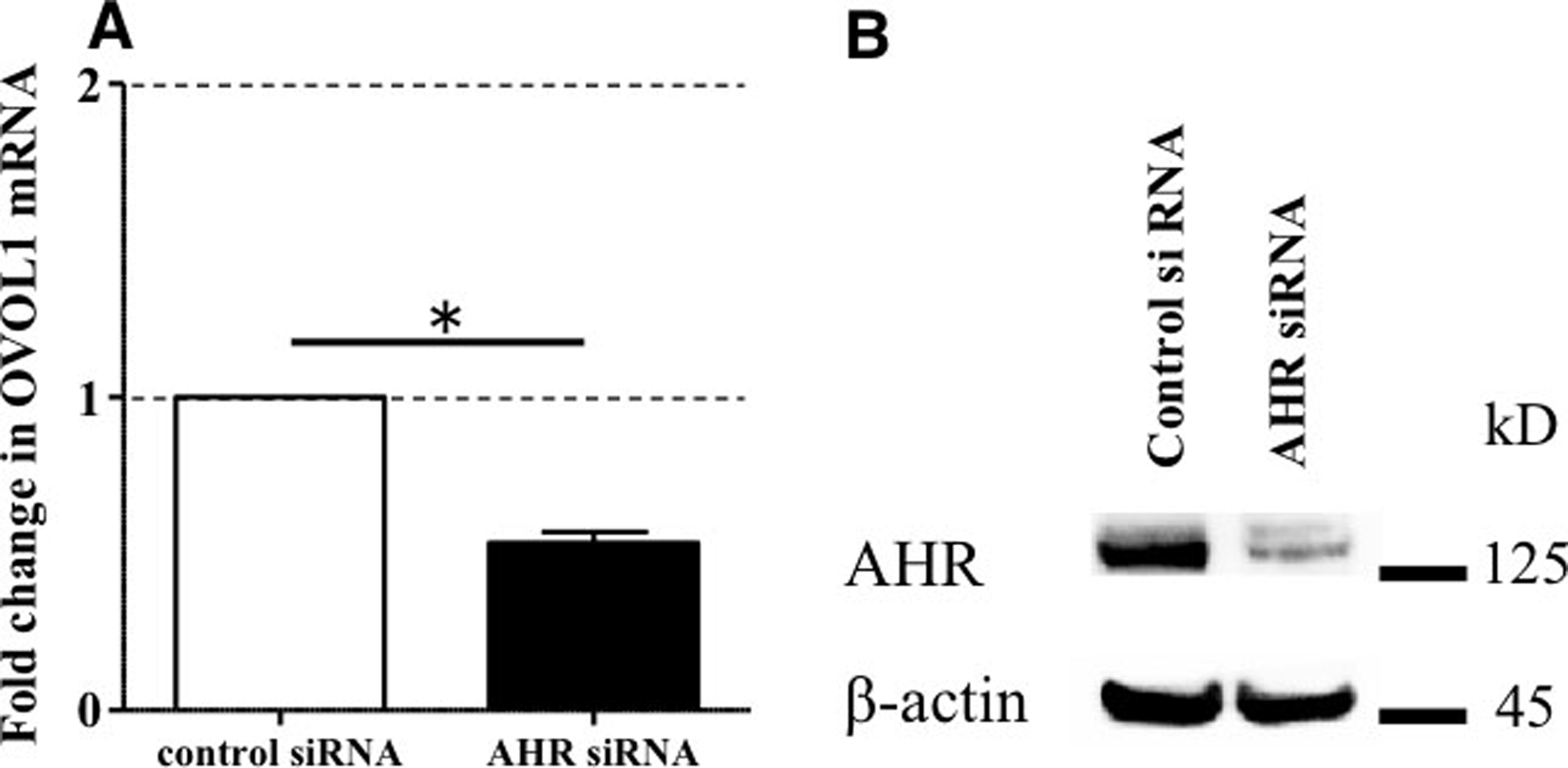

Supplement: Supplementary Figure S2 [file cddis2017322x2.tif]

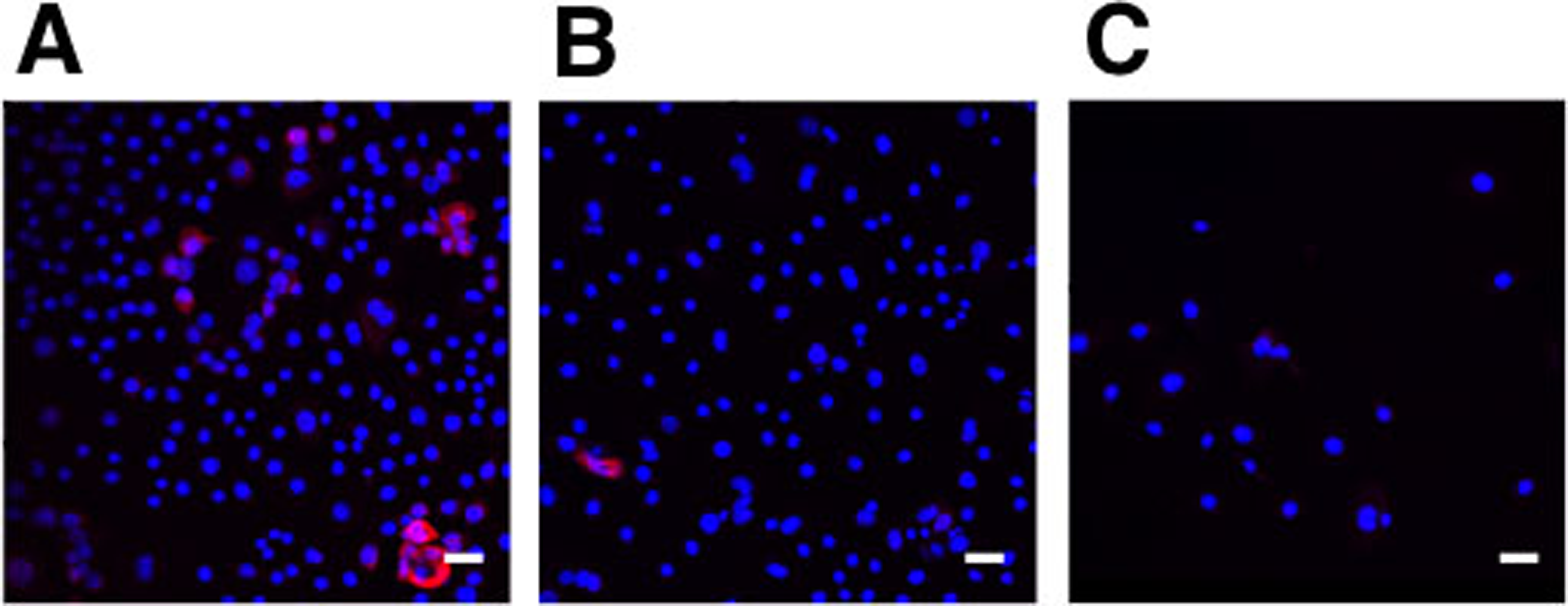

Supplement: Supplementary Figure S3 [file cddis2017322x3.tif]

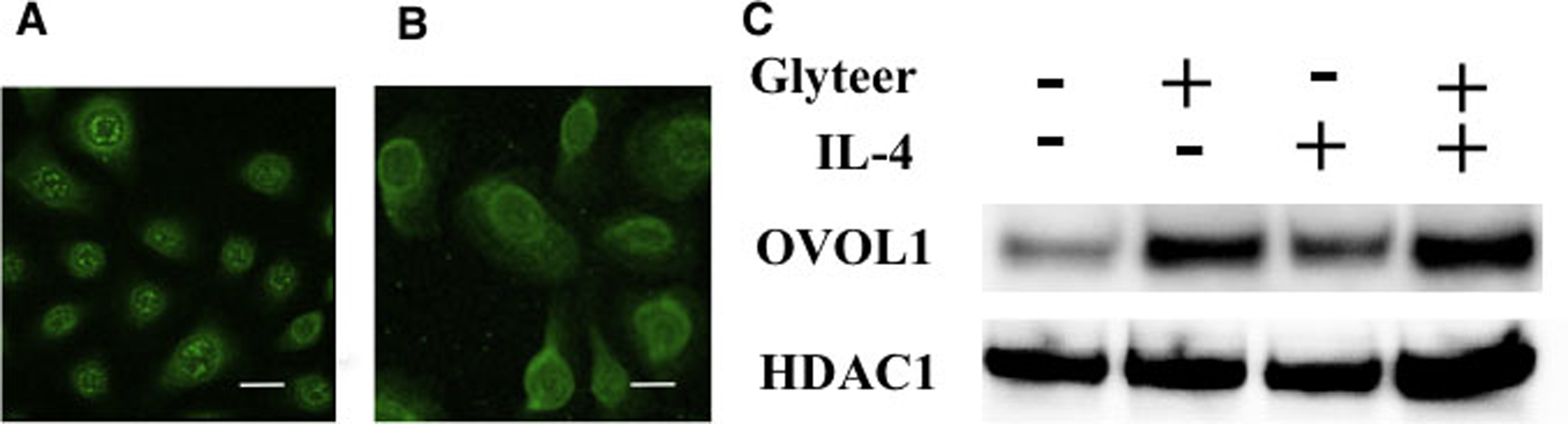

Supplement: Supplementary Figure S4 [file cddis2017322x4.tif]

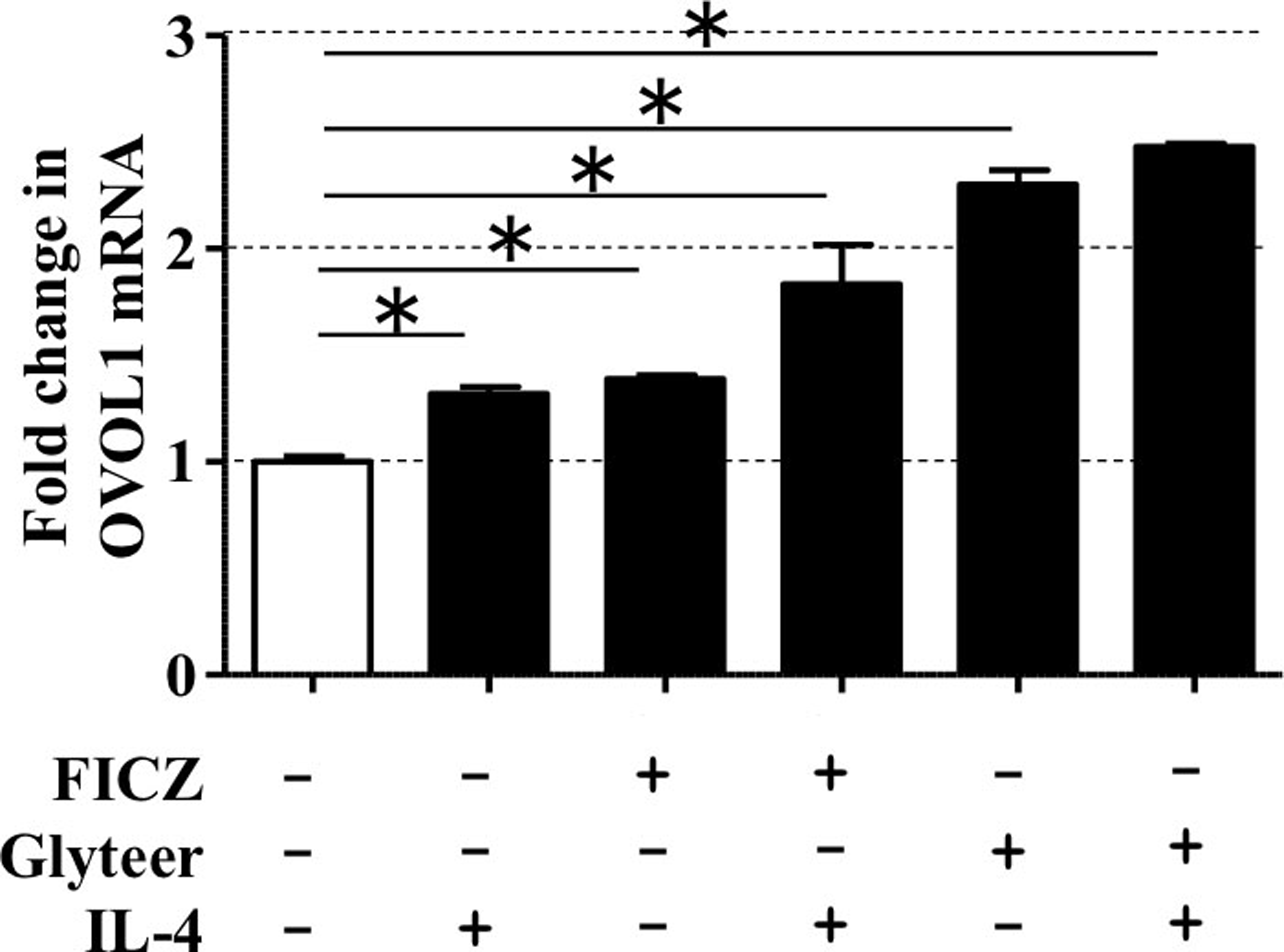

Supplement: Supplementary Figure S5 [file cddis2017322x5.tif]

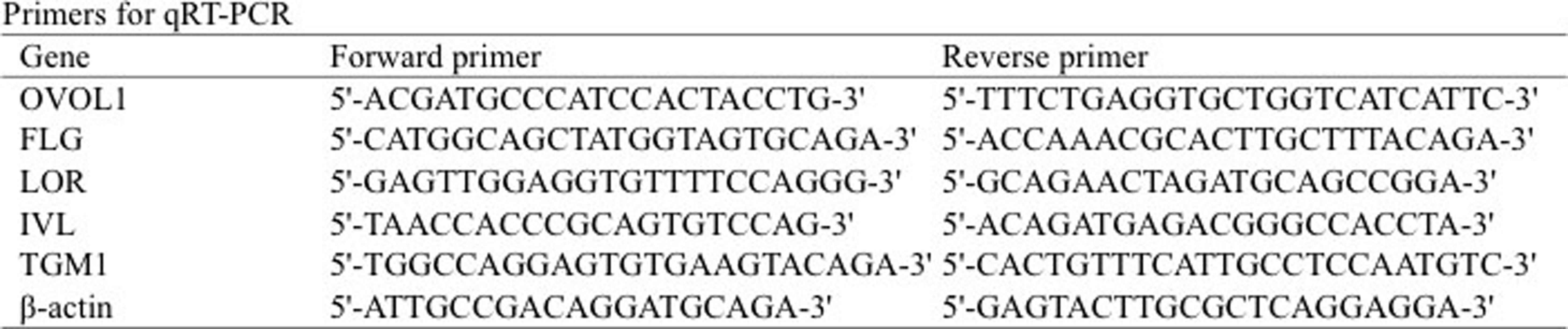

Supplement: Supplementary Table S1 [file cddis2017322x6.tif]
