# Supplementary material for: Aryl hydrocarbon receptor activation restores filaggrin expression via OVOL1 in atopic dermatitis
Source: Cell Death Dis. 2017 Jul 13;8(7):e2931–. doi: 10.1038/cddis.2017.322 (PMC5550867; doi:10.1038/cddis.2017.322)
Supplement: Supplementary Figure Legends [file cddis2017322x7.docx]

**Supplementary Figure S1** OVOL1 expression in OVOL1 OE NHEKs (A) or OVOL1 siRNA NHEKs (B) was analyzed by western blotting. The data are representative of experiments repeated three times with similar results. Expression of *LOR*, *IVL*, and *TGM1* in OVOL1 OE NHEKs (C) or OVOL1 siRNA NHEKs (D) was analyzed by qRT-PCR. Data are expressed as mean ± S. E. M.; n = 3 for each group; **P* < 0.05.

**Supplementary Figure S2** Expression of OVOL1 mRNA and protein in AHR siRNA-transfected NHEKs was analyzed by qRT-PCR (A) and western blotting (B). Data are expressed as mean ± S. E. M.; n = 3 for each group; **P* < 0.05 (A). The data are representative of experiments repeated three times with similar results (B).

**Supplementary Figure S3** NHEKs transfected with (A) control siRNA, (B) AHR siRNA, or (C) OVOL1 siRNA were treated with Glyteer (0.001 %) for 24 h. The NHEKs were stained with an anti-FLG antibody (primary antibody) and an Alexa Fluor 546-conjugated anti-mouse IgG antibody (secondary). The nucleus was counterstained with DAPI (blue). Confocal laser scanning images revealed that the upregulation of FLG (red) induced by Glyteer was observed in control siRNA-transfected NHEKs (A); this phenomenon was abrogated in AHR siRNA- (B) or OVOL1 siRNA-transfected NHEKs (C). The scale bar is 100 μm. The data are representative of experiments repeated three times with similar results.

**Supplementary Figure S4** NHEKs were treated with (A) Glyteer (0.001 %) or (B) IL-4 (10 ng/ml) and Glyteer (0.001 %) for 24 h. The NHEKs were stained with an anti-OVOL1 antibody (primary antibody) and an Alexa Fluor 488-conjugated anti-rabbit IgG antibody (secondary). The nucleus was counterstained with DAPI (blue). Confocal laser scanning images revealed that IL-4-mediated blockade of the OVOL1 nuclear translocation was overcome by treatment with FICZ. The scale bar is 100 μm. The data are representative of experiments repeated three times with similar results. (C) NHEKs were treated with Glyteer (0.001 %) in the absence or presence of IL-4 (10 ng/ml) for 18 h. Cellular nuclear protein was extracted using a biochemical subcellular fractionation technique. The OVOL1 levels in the nuclear protein fraction of NHEKs were evaluated by western blot analysis. The data are representative of experiments repeated three times with similar results.

**Supplementary Figure S5** NHEKs were treated with FICZ (100 nM) or Glyteer (0.001 %) with or without IL-4 (10 ng/ml) for 24 h. Expression of *OVOL1* was analyzed by qRT-PCR. Data are expressed as mean ± S. E. M.; n = 3 for each group; **P* < 0.05.

**Supplementary Figure S6** A list of primer sequences.
